# High-risk mammographic parenchymal patterns and anthropometric measures: a case–control study

**DOI:** 10.1038/sj.bjc.6690838

**Published:** 1999-12

**Authors:** E Sala, R Warren, J McCann, S Duffy, R Luben, N Day

**Affiliations:** Departments of Community Medicine, Clinical Gerontology, Strangeways Research Laboratory, Worts Causeway, Cambridge, CB1 8RN, UK; Cambridge and Huntingdon Breast Screening Service, Rosie Hospital, Cambridge, UK; MRC-Biostatistics UnitDepartment of Community Medicine, Institute of Public Health, Cambridge, UK

**Keywords:** mammographic parenchymal patterns, anthropometry, breast size

## Abstract

Mammographic parenchymal patterns are related to breast cancer risk and are also affected by anthropometric measure. We carried out a case–control study comprising 200 cases with high-risk (P2 and DY) mammographic parenchymal pattern and 200 controls with low-risk (N1 and P1) patterns in order to investigate the effect of body size and shape and breast size on mammographic patterns. Women in the highest quartile of body mass index (BMI) distribution were significantly less likely to have a high-risk pattern (odds ratio (OR) = 0.21, 95% confidence interval (CI) 0.08–0.52, *P*-value for trend = 0.001) compared to those in the lowest quartile. Relative to women with a waist to hip ratio (WHR) of less than 0.75, the OR of having a high-risk pattern in women with a WHR greater than 0.80 was 0.30 (95% CI 0.14–0.63). Breast size as measured by cup size was significantly and negatively related to high-risk pattern. Our study indicates that both BMI and WHR are negatively associated with high-risk patterns. However, both phenomena are associated with increased risk of breast cancer in post-menopausal women. This negative confounding of two positive risk factors means that the effect of parenchymal patterns on risk will tend to be underestimated when not adjusted for BMI and WHR and vice versa. Thus we may have underestimated the importance of BMI and mammographic parenchymal patterns in the past. Further studies are needed to determine a measure of parenchymal density that is independent of anthropometric measures and breast size. © 1999 Cancer Research Campaign


*British Journal of Cancer* (1999) **81**(7), 1257–1261
© 1999 Cancer Research Campaign
Article no. bjoc.1999.0838

### High-risk mammographic parenchymal patterns and anthropometric measures: a case–control study

**E Sala[1], R Warren[2], J McCann[1], S Duffy[3], R Luben[4] and N Day[5]**

Departments of [1]Community Medicine and [4]Clinical Gerontology, Strangeways Research Laboratory, Worts Causeway, Cambridge, CB1 8RN, UK; [2]Cambridge and Huntingdon Breast Screening Service, Rosie Hospital, Cambridge, UK; [3]MRC-Biostatistics Unit and [5]Department of Community Medicine, Institute of Public Health, Cambridge, UK

**Summary** Mammographic parenchymal patterns are related to breast cancer risk and are also affected by anthropometric measure. We carried out a case–control study comprising 200 cases with high-risk (P2 and DY) mammographic parenchymal pattern and 200 controls with low-risk (N1 and P1) patterns in order to investigate the effect of body size and shape and breast size on mammographic patterns. Women in the highest quartile of body mass index (BMI) distribution were significantly less likely to have a high-risk pattern (odds ratio (OR) = 0.21, 95% confidence interval (CI) 0.08–0.52, *P*-value for trend = 0.001) compared to those in the lowest quartile. Relative to women with a waist to hip ratio (WHR) of less than 0.75, the OR of having a high-risk pattern in women with a WHR greater than 0.80 was 0.30 (95% CI 0.14–0.63). Breast size as measured by cup size was significantly and negatively related to high-risk pattern. Our study indicates that both BMI and WHR are negatively associated with high-risk patterns. However, both phenomena are associated with increased risk of breast cancer in post-menopausal women. This negative confounding of two positive risk factors means that the effect of parenchymal patterns on risk will tend to be underestimated when not adjusted for BMI and WHR and vice versa. Thus we may have underestimated the importance of BMI and mammographic parenchymal patterns in the past. Further studies are needed to determine a measure of parenchymal density that is independent of anthropometric measures and breast size. © 1999 Cancer Research Campaign

**Keywords:** mammographic parenchymal patterns; anthropometry; breast size

Variations in morphologic features of the breast, such as the relative amounts of fat, connective tissue and epithelial tissue are visible on a mammogram and are referred to as the parenchymal pattern of the breast. These patterns were classified by Wolfe into four categories: N1, P1, P2 and DY (Wolfe, 1976*b*). The high-risk patterns P2 and DY are characterized by greater mammographic density. Certain mammographic parenchymal patterns have been positively associated with breast cancer risk (Wolfe, 1976*a*, 1976*b*; Saftlas and Szklo, 1987; Oza and Boyd, 1993; Sala et al, 1998).

There are conflicting data regarding the association of anthropometric measures with breast cancer on one hand and mammographic parenchymal patterns on the other. Several studies have shown that increased body weight and body mass index (BMI) are associated with a significant reduction in the percentage of women having a P2 or DY (P2/DY) pattern (Brisson et al, 1984; de Waard et al, 1984; Grove et al, 1985; De Stavola et al, 1990; Boyd et al, 1995, 1998; Salminen et al, 1998). However, overweight women have an elevated risk of developing post-menopausal breast cancer (Tornberg et al, 1988; van den Brandt et al, 1997). This has not been observed for premenopausal disease. The evidence is less consistent regarding the association between height and mammographic parenchymal pattern, although some studies found an increased frequency of P2 and DY patterns in the breasts of taller women (Brisson et al, 1984; Grove et al, 1985). Abdominal fat predominance (as measured by waist to hip ratio (WHR)) has been associated with a reduction in the proportion of women with P2/DY pattern (Beijerinck et al, 1991). Breasts of smaller size tend to have a high-risk mammographic parenchymal pattern (Brisson et al, 1984; Kato et al, 1995; Thurfjell et al, 1996; Salminen et al, 1998).

To obtain further information on these issues we examined the association between body size (as measured by weight, height and BMI), body shape (as measured by WHR) and breast size (as measured by cup size) in a case-control study nested within the European Prospective Investigation of Cancer-Norfolk (EPIC-Norfolk cohort) (Day et al, 1999).

*Received 13 April 1999*
*Revised 3 June 1999*
*Accepted 3 June 1999*

*Correspondence to:* E Sala

### MATERIALS AND METHODS

### Study population

Study participants were members of a cohort of women enrolled in the EPIC-Norfolk cohort (Day et al, 1999), who attended the prevalence screening round at the Norwich Breast Screening Programme between November 1989 and December 1997 and who did not have breast cancer diagnosed at the time of their prevalent screen. A case–control study was designed within this cohort.

Cases and controls were defined on the basis of mammographic parenchymal patterns. We examined the screening records of each woman. Mammograms of both breasts were collected. Both views

**Table 1** Odds ratio estimates for high-risk mammographic patterns according to anthropometric factors

| Anthropometric factors | Cases (P2+DY) | Controls (N1+P1) | OR | 95% CI | Trend test | OR[a] | 95% CI[a] | Trend test[a] |
|---|---|---|---|---|---|---|---|---|
| Weight (kg) | | | | | | | | |
| <55 | 22 | 10 | 1.00 | – | <0.0001 | 1.00 | – | <0.0001 |
| 55–64 | 87 | 68 | 0.58 | 0.24–1.40 | | 0.66 | 0.23–1.84 | |
| 65–74 | 63 | 52 | 0.54 | 0.22–1.31 | | 0.52 | 0.17–1.49 | |
| 75+ | 31 | 72 | 0.21 | 0.08–0.52 | | 0.23 | 0.08–0.64 | |
| Height (cm) | | | | | | | | |
| <155 | 31 | 28 | 1.00 | – | 0.72 | 1.00 | – | 0.46 |
| 155–159 | 52 | 60 | 0.76 | 0.39–1.48 | | 0.66 | 0.29–1.46 | |
| 160–164 | 65 | 61 | 0.94 | 0.50–1.76 | | 0.98 | 0.47–2.01 | |
| 165+ | 55 | 53 | 0.90 | 0.48–1.69 | | 1.14 | 0.52–2.45 | |
| BMI | | | | | | | | |
| <23 | 51 | 31 | 1.00 | – | <0.0001 | 1.00 | – | 0.001 |
| 23–24 | 58 | 41 | 0.83 | 0.44–1.53 | | 0.70 | 0.31–1.56 | |
| 25–29 | 75 | 84 | 0.48 | 0.26–0.89 | | 0.41 | 0.19–0.89 | |
| 30+ | 19 | 46 | 0.25 | 0.11–0.51 | | 0.21 | 0.08–0.52 | |
| Waist/hip ratio | | | | | | | | |
| <0.75 | 60 | 34 | 1.00 | – | <0.0001 | 1.00 | – | 0.002 |
| 0.75–0.79 | 68 | 57 | 0.63 | 0.34–1.15 | | 0.44 | 0.20–0.93 | |
| 0.80+ | 75 | 110 | 0.36 | 0.20–0.62 | | 0.30 | 0.14–0.63 | |
| Cup size | | | | | | | | |
| A | 31 | 9 | 1.00 | – | 0.001 | 1.00 | – | 0.002 |
| B | 73 | 82 | 0.20 | 0.07–0.54 | | 0.14 | 0.04–0.47 | |
| C | 61 | 61 | 0.20 | 0.07–0.55 | | 0.14 | 0.04–0.49 | |
| D+ | 26 | 42 | 0.11 | 0.03–0.32 | | 0.06 | 0.01–0.25 | |

[a]Adjusted for menopausal status, number of children, history of benign breast diseases, HRT, smoking and hysterectomy

(medio-lateral and cranio-caudal) were identified. All films were independently reviewed by two of the authors (ES and RW) to determine the mammographic parenchymal pattern as classified by Wolfe.

A total of 9484 women were identified by linking databases from EPIC-Norfolk cohort and the NHS Regional Breast Screening Programme for Norwich. Of these women, 445 had food diaries that had been entered into the EPIC-Norfolk database. We excluded 45 women because they were either diagnosed with a histologically verified breast cancer prior to or at the prevalent screen, or they did not respond to the screening invitations, or after an extensive search the screening mammogram or screening records were not located, or they had breast implants. Eligible cases were defined as women from the cohort with a P2/DY Wolfe's mammographic parenchymal pattern on the prevalence screen mammogram. In order for a case to be eligible, a mammogram had to be classified as P2/DY for both breasts and both views by the two readers. There was inter-reader disagreement for 17 women so these were excluded as potential cases. This left 383 women who satisfied the study criteria. A total of 203 women with P2/DY mammographic patterns were identified as cases.

For each case, we selected one control with a N1/P1 Wolfe's mammographic parenchymal pattern at the prevalence screen, matched to the case by date of birth (within 1 year) and date of prevalence screen (within 3 months). In order for a subject to be eligible as a control, a mammogram had to be classified as N1/P1 for both breasts and both views by the two readers. The readers disagreed for 13 women who were excluded as potential controls. A total of 167 women with N1/P1 Wolfe's mammographic patterns were identified as potential controls. Of these, only 141 could be individually matched for birth and prevalent screening date with the cases. The remaining 62 controls were identified among women with completed food diaries not yet entered on the database. Their diaries were entered afterwards. A total of 203 controls were identified.

### Risk factor data

The EPIC-Norfolk Health and Lifestyle Questionnaire provided information on the risk factors of interest. These included the personal and family history for benign breast diseases and cancer, menstrual factors and menstrual history, reproductive history, oral contraception and hormone replacement therapy, physical activity, smoking, and anthropometric measures such as weight, height, WHR and cup size. Menstrual status was defined as having had no menstrual periods for at least 6 months.

### Statistical methods

Data were analysed by conditional logistic regression, which takes into account the matching of controls to cases and produces odds ratio (OR) estimates of relative risk and their 95% confidence intervals (CI) (Breslow and Day, 1980). Odds ratios were adjusted for those factors which were previously found (details available from the authors) to be associated with high-risk mammographic parenchymal patterns (menopausal status, number of children, history of benign breast disease, hormone replacement therapy use, smoking and hysterectomy in the study as a whole; number of children, smoking, hysterectomy for post-menopausal women only).

### RESULTS

The age range of cases was 46–73 years old; for controls it was identical. Twenty-three per cent of the cases and 13% of the controls were pre- or peri-menopausal.

 *© 1999 Cancer Research Campaign*

**Table 2** Odds ratio estimates for high-risk mammographic patterns according to anthropometric factors in post-menopausal women

| Anthropometric factors | Cases (P2+DY) | Controls (N1+P1) | OR | 95% CI | Trend test | OR[a] | 95% CI[a] | Trend test[a] |
|---|---|---|---|---|---|---|---|---|
| Weight (kg) | | | | | | | | |
| <55 | 15 | 8 | 1.00 | – | 0.002 | 1.00 | – | 0.004 |
| 55–64 | 64 | 54 | 0.94 | 0.33–2.59 | | 0.89 | 0.30–2.65 | |
| 65–74 | 46 | 42 | 0.78 | 0.27–2.18 | | 0.66 | 0.21–2.03 | |
| 75+ | 23 | 60 | 0.32 | 0.11–0.89 | | 0.29 | 0.09–0.87 | |
| Height (cm) | | | | | | | | |
| <155 | 25 | 25 | 1.00 | – | 0.7 | 1.00 | – | 0.3 |
| 155–159 | 37 | 51 | 0.77 | 0.34–1.70 | | 0.76 | 0.31–1.81 | |
| 160–164 | 48 | 45 | 1.20 | 0.58–2.50 | | 1.31 | 0.59–2.89 | |
| 165+ | 38 | 43 | 0.94 | 0.44–2.00 | | 1.28 | 0.54–2.97 | |
| BMI | | | | | | | | |
| <23 | 34 | 24 | 1.00 | – | 0.005 | 1.00 | – | 0.004 |
| 23–24 | 40 | 29 | 1.20 | 0.54–2.69 | | 0.88 | 0.35–2.21 | |
| 25–29 | 59 | 73 | 0.69 | 0.33–1.41 | | 0.57 | 0.25–1.31 | |
| 30+ | 15 | 38 | 0.33 | 0.13–0.78 | | 0.22 | 0.08–0.58 | |
| Waist/hip ratio | | | | | | | | |
| <0.75 | 41 | 27 | 1.00 | – | 0.002 | 1.00 | – | 0.003 |
| 0.75–0.79 | 54 | 45 | 0.56 | 0.24–1.27 | | 0.51 | 0.20–1.30 | |
| 0.80+ | 53 | 91 | 0.29 | 0.13–0.65 | | 0.26 | 0.10–0.63 | |
| Cup size | | | | | | | | |
| A | 20 | 8 | 1.00 | – | 0.066 | 1.00 | – | 0.03 |
| B | 55 | 68 | 0.25 | 0.08–0.79 | | 0.13 | 0.02–0.54 | |
| C | 44 | 50 | 0.31 | 0.09–0.98 | | 0.16 | 0.03–0.64 | |
| D+ | 21 | 33 | 0.16 | 0.04–0.62 | | 0.08 | 0.01–0.42 | |

[a]Adjusted for number of children, smoking, hysterectomy.

Table 1 shows the unadjusted and adjusted OR estimates for Wolfe's high-risk mammographic parenchymal patterns and different anthropometric factors in the total study population. The odds of having a high-risk pattern in women who weighed more than 75 kg was approximately one-fifth that in women who weighed less than 55 kg (OR = 0.23; 95% CI 0.07–0.64). A significant trend with increasing weight was observed ($P < 0.0001$). The above findings persisted when the analysis was limited to post-menopausal women (OR = 0.29; 95% CI 0.09–0.87) (Table 2).

Height was not related to mammographic parenchymal patterns in the total study population (Table 1). Post-menopausal women who were 160 cm or taller were at greater risk of having a high-risk mammographic pattern compared to post-menopausal women shorter than 160 cm, but statistical significance was not reached (Table 2).

BMI was strongly and inversely associated with high-risk patterns. Relative to the lowest quartile, women in the highest quartile of the BMI distribution were significantly less likely to have a high-risk pattern (OR = 0.21, 95% CI 0.08–0.52). There was a significant trend across the quartiles of BMI ($P$-value = 0.001). Similar results were obtained when the analysis was confined to post-menopausal women (Table 2).

Relative to women with a WHR of less than 0.75, the OR of having a high-risk mammographic pattern in women with a WHR of greater than 0.80 was 0.30 (95% CI 0.14–0.63). The protective effect of WHR persisted when the analysis was limited to post-menopausal women (OR = 0.26; 95% CI 0.10–0.63) (Table 2).

Breast size, as measured by cup size, was significantly and negatively related to high-risk pattern in the total study population (OR = 0.06; 95% CI 0.01–0.25) (Table 1). Among post-menopausal women the unadjusted association between breast size and high-risk patterns was not statistically significant. After adjustment, however, the association achieved significance (OR = 0.08; 95% CI 0.01–0.42) (Table 2).

**Table 3** Wolfe mammographic patterns distribution according to BMI

| Wolfe's parenchymal pattern | BMI categories (kg m$^{-2}$) | | | |
|---|---|---|---|---|
| | <25 *n* (%) | 25–30 *n* (%) | >30 *n* (%) | N |
| N1 | 30 (17) | 38 (24) | 24 (37) | 92 |
| P1 | 42 (23) | 46 (29) | 22 (34) | 110 |
| P2 | 69 (38) | 59 (37) | 18 (28) | 146 |
| DY | 40 (22) | 16 (10) | 1 (1) | 57 |
| N | 181 | 159 | 65 | 405 |

### DISCUSSION

In this study, we found strong inverse associations between weight and BMI and mammographic parenchymal patterns of breast tissue as classified by Wolfe. Other studies have reported a relationship between body weight and mammographic parenchymal pattern similar to our own (Brisson et al, 1984; de Waard et al, 1984; Grove et al, 1985; De Stavola et al, 1990; Boyd et al, 1995, 1998; Salminen et al, 1998). Boyd et al (1998) found that, in premenopausal women, weight and BMI were negatively correlated with the area of dense tissue.

With respect to height, we found a weak positive association with high-risk parenchymal patterns when the analysis was confined to post-menopausal women only. Other studies have found an increased frequency of P2 and DY patterns in the breasts of taller women (Brisson et al, 1984; Grove et al, 1985), but this

*© 1999 Cancer Research Campaign*

relation was not as strong as that between body weight and parenchymal pattern.

We found a strong inverse relationship between WHR and parenchymal patterns. Beijerinck et al (1990) also found that high WHR is associated with the incidence of favourable (N1, P1) mammographic parenchymal patterns. In our study breast size, as measured by cup size, was significantly and inversely associated with mammographic parenchymal patterns. Our finding is supported by other studies although different modalities were used to measure the size of the breast (Brisson et al, 1984; Kato et al, 1995; Thurfjell et al, 1996; Salminen et al, 1998).

Our study design minimized the opportunity for bias to influence our findings. Systematic error in the assessment of mammograms was avoided since reading was done without knowledge of risk factor data. Weight, height, waist and hip were measured by a nurse involved in the EPIC-Norfolk cohort following an agreed protocol (Day et al, 1999) thus eliminating recall bias. Furthermore, the associations observed are unlikely to be explained by the confounding effect of other possible breast cancer risk factors since we adjusted for these in the analysis.

It seems that the direct association of weight, WHR and breast size to breast cancer risk is not due to associations of these factors with high-risk mammographic parenchymal patterns. Breast cancer risk increases with increasing weight (Tornberg et al, 1988; van den Brandt et al, 1997), WHR (Schapiro et al, 1990; Sellers et al, 1992) and, possibly, breast size (Kato et al, 1995; Scutt et al, 1997). However, excess body weight, high WHR and large breasts are all associated with low-risk parenchymal patterns which, in turn, relate to a decreased risk for breast cancer. Brisson et al (1984) suggested that adjusting for weight and height is important when evaluating the relationship between mammographic parenchymal pattern and breast cancer risk since these may be important confounding factors. Our findings indicate that WHR and breast size are also confounders. Our study indicates that obesity, as represented by high BMI or WHR, is negatively associated with high-risk mammographic parenchymal patterns. However, both phenomena are associated with increased risk of breast cancer in post-menopausal women. This negative confounding of two positive risk factors means that the effect of parenchymal patterns on risk will tend to be underestimated when not adjusted for some measure of obesity and vice versa. This in turn suggests that in the past, we may have underestimated the importance of BMI and of mammographic parenchymal patterns in assessing the breast cancer risk. For example, previously reported studies that did not adjust for body size and shape (Wolfe, 1976*a*; Saftlas and Szklo, 1987; Oza and Boyd, 1993) might have underestimated the true association between mammographic parenchymal patterns and breast cancer.

An interesting point is whether it is possible to determine a measure of parenchymal density that is independent of body habitus and breast size. The fact that Wolfe classification system depends on percentages of the breast with dense parenchyma implies that, in this system, an association with breast size is inevitable. It may also be inevitable that a higher BMI means more adipose tissue generally and more fatty replacement in the breast. Table 3 shows the four individual Wolfe patterns according to BMI. Clearly lower proportions of both high-risk groups P2 and DY are associated with high BMI, but the effect is less pronounced for P2. Thus it may be that some aspects of the P2 pattern (e.g. nodular densities) are not related to breast size or obesity.

### ACKNOWLEDGEMENTS

We thank Anglia and Oxford Health Authority, R & D Programme for funding this study. We are most grateful to Dr Graham Hurst, director of Norwich Breast Screening Unit. We also thank all the staff of Norwich Breast Screening Unit for their invaluable help during data collection. We also thank the staff of EPIC-Norfolk for their contribution to the study.

### REFERENCES

Beijerinck D, van Noord PAH, Seidell JC, den Tonkelaar I, Rombach JJ and Brunning PF (1991) Abdominal fat predominance in women is associated with a decreased prevalence of the high risk P2, DY mammographic breast patterns. *Int J Obesity* **15**: 89–93

Boyd NF, Connelly P, Byng J, Yaffe M, Drapper H, Little L, Jones D, Martin LJ, Lockwood GA and Tritchler D (1995) Plasma lipids, lipoproteins, and mammographic densities. *Cancer Epidemiol Biomarkers Prev* **4**: 727–733

Boyd NF, Lockwood GA, Byng JW, Little L, Yaffe and Tritchler D (1998) The relationship of anthropometric measures to radiological features of the breast in premenopausal women. *Br J Cancer* **78**: 1233–1238

Breslow NE and Day NE (1980) *Statistical Methods in Cancer Research*. Vol. 1. *The Analysis of Case–control Studies*. IARC Scientific Publications: Lyon, France

Brisson J, Morrison AS, Copans DB, Sadowsky N, Kalisher E, Twadle JA, Meyer JE, Henschke CI and Cole P (1984) Height and weight, mammographic features of breast tissue, and breast cancer risk. *Am J Epidemiol* **119**: 371–381

Day N, Oakes S, Luben R, Khaw KT, Bingham S, Welch A and Wareham NJ (1999) EPIC in Norfolk: study design and characteristics of the cohort. *Br J Cancer* (in press)

De Stavola B, Gravelle IH, Wang DY, Allen DS, Bulbrook RD, Fentiman IS, Hayward JL and Chaudary MC (1990) Relationship of mammographic parenchymal patterns with breast cancer risk factors and risk of breast cancer in a prospective study. *Int J Epidemiol* **19**: 247–254

de Waard F, Rowbach II, Collette HJA and Beinard S (1984) Breast cancer risk associated with reproductive factors and breast parenchymal patterns. *J Natl Cancer Inst* **72**: 1277–1282

Grove JS, Goodman MJ, Gilbert FI and Mi MP (1985) Factors associated with mammographic pattern. *Br J Radiol* **58**: 21–25

Kato I, Benairt C, Bleich A, Su S, Kim M and Toniolo PG (1995) A nested case–control study of mammographic patterns, breast volume, and breast cancer. *Cancer Causes Control* **6**: 431–438

Oza AM and Boyd NF (1993) Mammographic parenchymal patterns: a marker of breast cancer risk. *Epidemiol Rev* **15**: 196–208

Saftlas AF and Szklo M (1987) Mammographic parenchymal patterns and breast cancer risk. *Epidemiol Rev* **9**: 146–174

Sala E, Warren R, McCann J, Duffy S, Day N and Luben R (1998) Mammographic parenchymal patterns and mode of detection: implications for the breast screening programme. *J Med Screen* **5**: 180–185

Salminen T, Hakama M, Heikkila M and Saarenmaa I (1998) Favourable change in mammographic parenchymal patterns and breast cancer risk factors. *Int J Cancer* **78**: 410–414

Schapiro DV, Kumar NB, Lyman GH and Cox CE (1990) Abdominal obesity and breast cancer. *Ann Intern Med* **112**: 182–186

Scutt D, Manning JT, Whitehouse GJ, Leinster SJ and Massey CP (1997) The relationship between breast assymetry, breast size and the occurrence of breast cancer. *Br J Radiol* **70**: 1017–1021

Sellers TA, Kushi LH, Potter JD, Kaye SA, Nelson CL, McGovern PG and Folsom AR (1992) Effect of family history, body-fat distribution, and reproductive factors on the risk of post-menopausal breast cancer. *N Engl J Med* **326**: 1323–1329

Thurfjell E, Hsieh C-C, Lipworth L, Ekbom A, Adami HO and Trichopoulos D (1996) Breast size and mammographic pattern in relation to breast cancer risk. *Eur J Cancer Prev* **5**: 37–41

Tornberg SA, Holm LE and Cartensen JM (1988) Breast cancer risk in relation to serum cholesterol, serum beta-lipoprotein, height, weight, and blood pressure. *Acta Oncol* **27**: 31–37

van den Brandt P, Dirx MJM, Ronckers CM, van den Hoogen P and Goldbohm AR (1997) Height, weight, weight change, and post-menopausal breast cancer risk: the Netherlands Cohort Study. *Cancer Causes Control* **8**: 39–47

*© 1999 Cancer Research Campaign*

Wolfe JN (1976*a*) Breast patterns as an index of risk for developing breast cancer. *Am J Roentgen* **126**: 1130–1139

Wolfe JN (1976*b*) Risk for breast cancer development determined by mammographic parenchymal patterns. *Cancer* **37**: 2486–2492

*© 1999 Cancer Research Campaign*
